# Comparison of Thawing Treatments on Quality, Microbiota, and Organoleptic Characteristics of Chicken Meat Fillets

**DOI:** 10.1021/acsomega.3c03385

**Published:** 2023-07-10

**Authors:** Muhammad Waqas Arshad, Muhammad Rizwan Tariq, Shinawar Waseem Ali, Zunaira Basharat, Zujaja Umer, Gulzar Ahmad Nayik, Seema Ramniwas, Abeer S. Aloufi, Sulaiman Ali Alharbi, Mohammad Javed Ansari, Sezai Ercisli

**Affiliations:** † Department of Food Sciences, University of the Punjab, Quid-i-Azam Campus, Lahore 54590, Pakistan; ‡ Department of Food Science & Technology, Govt. Degree College Shopian, J&K 192303, India; § University Centre for Research and Development, Chandigarh University, Gharuan, Mohali, Punjab 140413, India; ∥ Department of Biology, College of Science, Princess Nourah bint Abdulrahman University, P.O. Box 84428, Riyadh 11671, Saudi Arabia; ⊥ Department of Botany and Microbiology, College of Science, King Saud University, P.O. Box 2455, Riyadh 11451, Saudi Arabia; # Department of Botany, Hindu College Moradabad (Mahatma Jyotiba Phule Rohilkhand University Bareilly), Uttar-Pradesh 244001, India; ¶ Department of Horticulture, Faculty of Agriculture, Ataturk University, 25240 Erzurum, Türkiye; ∇ HGF Agro, Ata Teknokent, TR-25240 Erzurum, Türkiye; ▼ Al-Waili Foundation for Science, Queens, New York 11418, United States

## Abstract

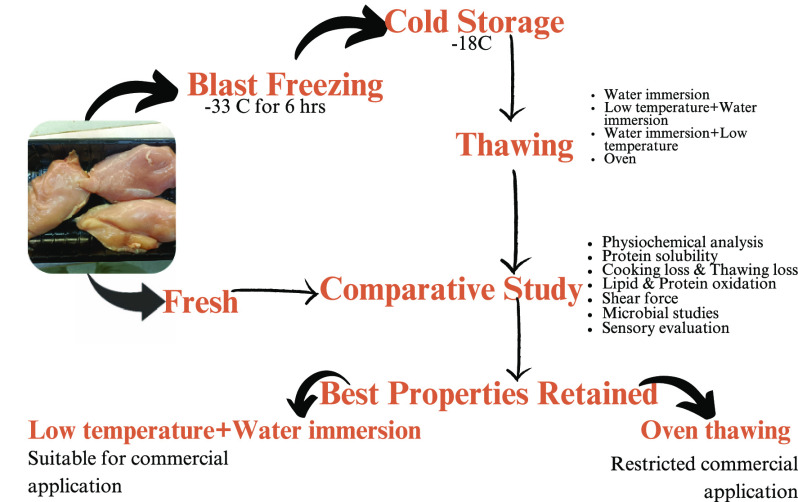

The current research attempted to evaluate the impact of various thawing techniques (R_0_: control group, R_1_: water immersion thawing, R_2_: low-temperature thawing, R_3_: combined thawing, water thawing then low-temperature thawing, R_4_: combination thawing, low temperature thawing then water thawing, and R_5_: oven thawing) on the quality, microbiota, and organoleptic characteristics of chicken meat fillets. The findings showed that moisture content varied from 74.43 to 72.33%; thawing loss peaked in R_1_ at 4.66%, while it was minimum in R_5_ at 2.10%. Lipid content varied from 1.09% in R_0_ to 1.03% in R_5_, while protein content varied from 22.06% in R_0_ to 23.10% in R_1_. The values of shear force, protein, and lipid oxidation increased for all treatments compared to control, ranging from 7.94 N to 9.54 N, 0.99–1.21 nm/mg protein, and 0.74–1.15 mg MDA/Kg, respectively. On the other hand, pH (5.94 in R_4_) and protein solubility (238.63 mg/g in R_1_) were decreased in contrast to the control group (6.08 and 298.27 mg/g). In association with different methods, R_5_ and R_2_ showed minimal thawing loss and the highest lipid and protein oxidation rates. However, R_3_ showed reduced shear force and lipid oxidation comparatively. TPC was significantly (*P* < 0.05) increased in both R_2_ and R_1_. Sensory evaluation indicated that R_3_ and R_2_ showed better color and taste, while R_1_ showed minimum scores for organoleptic attributes. R_0_, R_3_, and R_5_ obtained a higher sensory score_,_ whereas R_1_, R_2_, and R_4_ showed a lower score. However, R_5_ exhibited better results in close association with the control group (R_0_). Hence, it can be concluded that freezing and subsequent thawing decrease the quality of chicken fillets due to the time required for thawing. In the present study, the best quality of chicken fillets was retained by R_3_ and R_5_ due to their reduced thawing periods.

## 1 Introduction

Chicken meat is an essential component of the human diet due to its high protein and low fat content.^1^ Additionally, the extraordinary sensory properties of chicken meat are a primary reason for its increased demand among consumers and the processing industry. However, this type of meat is a perishable commodity with high moisture, contributing to very low shelf stability. In order to retain the quality and nutrition of meat, it is mandatory to preserve it. Freezing is frequently used to enhance the shelf life of meat with minimal quality or sensory deterioration. In the meat industry, freezing and thawing are widely used to enhance the shelf life of chicken meat or its products.

Poultry meat consumption has evolved along with population growth and is now regarded as a significant part of the human diet.^2,3^ As consumers increase daily, the demand for safe poultry products also increases, mandating the requirement for quality control and quality assessment protocols.^4^ Freezing is preferred for the preservation of meat and meat products in the international export market as it extends the shelf life of meat while maintaining quality characteristics over a long storage period. The projected value of the global frozen meat industry is around 13 billion USD per annum.^5,6^

Thawing frozen meat is a mandatory operation prior to its further processing or subsequent cooking. However, thawing can severely affect the meat’s quality in terms of moisture loss (drip loss), color deterioration, flavor depreciation, textural variations, microbial proliferation, and oxidative degradation of proteins and lipids.^7−9^ Furthermore, freezing and thawing are complex methods involving heat transfer and successive chemical changes, ultimately influencing the organoleptic attributes of the meat and meat products.^10^ The meat quality will inevitably deteriorate during thawing. However, this loss can be mitigated or even eliminated by employing the appropriate thawing techniques.

Conventional thawing methods such as air thawing, refrigeration thawing, or cold-water thawing result in poor-quality meat, primarily attributed to the prolonged stay in the danger zone and significant temperature differences.^8,11^ On the other hand, novel thawing techniques include high-pressure, Ohmic, and microwave thawing. These techniques significantly improved thawing rates with reduced quality defects, but certain limitations are still associated with these modern techniques.^12−14^ Li et al. proposed the application of combined thawing techniques to attain high-quality meat with diminished deteriorative losses during thawing.^15^

However, limited investigations have been made on various combinations of thawing techniques and their subsequent effect on meat quality. Therefore, the proposed study was designed to compare the various combined thawing techniques and their consequent impact on chicken meat quality indicators, including the analyses of (a) physiochemical parameters, (b) microbiological parameters, (c) oxidative parameters, and (d) organoleptic characteristics of chicken breast fillets.

## 2 Material and Methods

### 2.1 Raw Material Procurement and Sample Preparation

The freshly slaughtered chicken breast fillet was procured from a local primary processing unit in Lahore, Pakistan. The batch of birds was 39 days old, and samples were collected within 8 h of slaughtering. Each of the pectoralis was obtained and then transported to the laboratory of a primary processing unit in the ice box within 1 h of slaughtering. In the laboratory sample, preparation was done by removing the extra fatty tissue and connective tissues, or fascia, present on the skin of the breasts. After trimming off the extra-muscular portion, pieces were sliced with dimensions of length 7 cm, width 8 cm, and height 1.5 cm. The breast fillets were packed in LDPE bags with the dimensions of a length of 16 cm, a width of 29 cm, and a height of 0.003 cm and sealed with an electrical sealer acquired from a local market. R_0_ was designated as the control sample for examination under fresh, unfrozen conditions among these prepared samples. In contrast, the remaining samples were packed and shifted to a blast freezer with a circulating air temperature of −33 °C for about 6 h. Afterward, samples were transferred to the cold storage at a temperature of −18 °C for subsequent investigations according to the thawing protocol reported previously.^16^ In a single investigation, frozen samples were thawed to compute results, whereas unfrozen samples were taken as controls. For each treatment, there were three replicates, and all analysis was performed in triplicate.

### 2.2 Thawing Plan

Thawing was conducted in controlled conditions to achieve an internal core temperature of 0–2 °C. During thawing, core temperature was continuously evaluated using a probe meat thermometer following the method of Thanonkaew et al.^17^ Thawing was accomplished by following the thawing plan elucidated in (Table 1). For water thawing (R_1_), samples were retained in the temperature-controlled water tank for about 30 min, and the water-to-meat ratio was 1:40. On the other hand, low-temperature thawing (R_2_) was performed for almost 240 min to attain the desired internal core temperature. For all treatments (R_2_, R_3_, and R_4_), low-temperature thawing was carried out in a humidity and temperature control incubator. R_3_ and R_4_ samples were thawed collectively for 130 min and 37 min, respectively. Oven thawing was performed using the domestic microwave oven (Model DW 162 HZP, Dawlance, Pakistan) until the core temperature of 0–2 °C was achieved.

**Table 1:** Thawing Plan for Frozen Chicken Meat Fillets

treatments	thawing method	description
R_0_	fresh chicken fillet (Control)	unfrozen
R_1_	water immersion thawing	15 °C for 30 min
R_2_	low temperature thawing	0–4 °C for 240 min
R_3_	combined thawing (low temperature water thawing)	4 °C for 240 + 7 min
R_4_	combined thawing (water thawing + low temperature thawing)	15 °C for 30 + 22 min
R_5_	oven thawing	until 0–2°C achieved

### 2.3 Physiochemical Analysis

AOAC^18^ methods were used to evaluate moisture content (AOAC Method no. 948.12), crude Protein (AOAC Method no. 2011.04), and crude fat content (AOAC Method no. 948.22) in control and thawed samples. pH measurement of samples was performed utilizing an electronic and digital pH meter (HANNA-instrument, USA). The pH of all samples was recorded using the method of Wei et al^19^ All samples weighing 0.5 g were transferred to a centrifuged tube with 4.5 mL of pure water and agitated, and the pH was recorded against a stabilized reading.

### 2.4 Determination of Protein Solubility

The solubility of proteins was determined according to the method of Nahar et al.^20^ with slight modifications. To calculate the total amount of soluble proteins, a 0.30 g sample was taken and mixed with 6 mL of 0.1 M potassium phosphate buffer that had been precooled on ice (pH of the buffer was 7.1, 1.1 molarity potassium iodide). For extraction, chopped meat samples were mixed with ice in a shaker at 6000 rpm for 25 s for 12 h for extraction, followed by centrifugation at 1600*g* for 20 min at a temperature of 4 °C. Protein solubility (S) was determined by using the supernatant obtained after centrifugation and computed using the equation below.
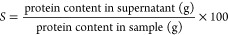


### 2.5 Determination of Cooking Loss and Thawing Loss

Cooking loss was estimated using the methods of Sunantha and Saroat.^21^ In particular, treatment samples (10 g) were air fried at 200 °C for 12–16 min and then allowed to cool down at 25 °C for 2–3 min. Subsequently, the filter paper was used to dry the surface of the fried samples; then, these were weighed, and the mass difference was calculated as cooking loss. Thawing loss was determined as the difference in sample weight before and after thawing. The sample weight after thawing was recorded after removing visible moisture from the meat surface through a water-soaking cloth. Thawing loss and cooking loss were calculated using the following formulas
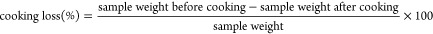

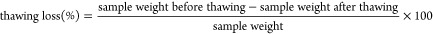


### 2.6 Determination of Lipid and Protein Oxidation

The thiobarbituric acid reactive substances (TBARS) were evaluated to foresee the lipid oxidation in thawed breast samples. TBARS were quantified with minor modifications according to Mashau et al.^22^ In brief, ethanolic extracts of thawed samples were mixed with thiobarbituric acid and centrifuged at 3000*g* for 15 min. Samples were heated at 95 °C for 60 min in a water bath, followed by cooling at 25 °C. The absorbance of samples was measured at 532 nm through the Tecan Sunrise spectrophotometer (Austria).

The carbonyl content in the thawed samples was measured using the method of Chen et al.^23^ The sample solution (2 mg/mL, 1 mL) was reacted with an equal volume of 2,4-dinitrophenylhydrazine (DNPH) (10 mM, 2 M HCl) in the dark for 60 min, with the mixture being agitated every 10 min. Afterward, the mixture was added to 20% trichloroacetic acid (1 mL), set down for 10 min, and centrifuged at 4 °C (1000*g* for 5 min). After removing the supernatant, 1 mL of ethanol/ethyl acetate (1:1, *v*/*v*) solution was added to the precipitate and centrifuged (4 °C, 1000*g*) for 5 min. To eliminate the extra DNPH, the precipitate was washed thrice with ethanol: ethyl acetate. Subsequently, the precipitate was mixed with 6 M guanidine hydrochloride (3 mL, 2 M HCl), kept at 37 °C for 15 min, and centrifuged at 4 °C (1000*g* for 3 min). The absorbance of the supernatant was subsequently measured at 370 nm using a spectrophotometer. The sample solution (2 mg/mL, 1 mL) was mixed with 2 M HCl (1 mL) as the blank sample. The protein hydrazones’ absorption coefficient of 22,000 M^–1^ cm^–1^was used to compute the carbonyl content, which was expressed as nanomoles of DNPH fixed per mg of protein.

### 2.7 Determination of Shear Force

Shear force determination was performed as reported by Baublits et al.^6^ After the cooking loss assessment, every sample was sliced longitudinally into a rectangular shape with the common kitchen knife. A cube was made from each sample with dimensions of 1 × 1 × 3 cm. All the samples were subjected to shear force measurement through the digital tenderness measurement instrument (Model C-LM3B, China). The highest shear force point was logged and recorded as a shear force value, and the readings were expressed in Newton (N).

### 2.8 Microbiological Analysis

Thawed samples’ microbial safety was assessed by performing a total plate count (TPC), *Coliform*, and *Listeria monocytogenes* examination for each treatment. Microbial proliferation was achieved using plate count agar, and blood agar media was used for TPC, *Coliform,* and *L. monocytogenes*.

#### 2.8.1 Media Preparation

Respective microbial media were prepared according to the formulation mentioned in (Table 2). After complete dissolution, the media were autoclaved for 20 min at 121 °C and 15 psi pressure. Autoclaved media were allowed to cool down and poured into sterilized Petri plates in a sterilized chamber.

**Table 2:** Formulation of Microbial Media

plate count agar	
ingredients	quantity
trypton	1.6 g
yeast extract	0.8 g
glucose	0.32 g
agar	4.8 g
distilled water	312.48 mL
Lactose Broth	
peptone	5 g
lactose	5 g
beef extract	3 g
distilled water	1000 mL
Blood Agar Media	
nutrient agar	7 g
distilled water	320 mL

#### 2.8.2 Sample Preparation

Samples were prepared in accordance with Kaewthong et al.^24^ 10 g of meat fillets were taken for each treatment and mixed with 90 mL of peptone solution. The peptone solution consisted of 0.1% (w/v) and 0.9% (w/v) NaCl. Approximately three dilutions were prepared from every sample, and then the prepared samples were inoculated onto the Petri dishes and incubated. Plate-count agar-containing Petri plates were incubated at 27 °C for 24 h.

#### 2.8.3 Presumptive Test

For coliforms and Listeria, presumptive tests were performed. Lactose broth (5 mL) was poured into 5 test tubes individually for all the thawed chicken breast samples, Stoppard utilizing aluminum foils, and incubated at 32 °C for 48 h. For Listeria, blood agar enriched with 0.05% potassium tellurate was used. Media-prepared slants and thawed samples were inoculated onto slants and stored at 37 °C for 24 h after a clod treatment at 4 °C.

#### 2.8.4 Confirmatory Test

Coliform conformation was done after taking the colonies from lactose broth and inoculating them on eosin-methylene blue agar. Dark colonies along, with a metallic sheen, confirmed the presence of *Coliform*.^25^ For *L. moncytogenes*, colonies were transferred onto slides and observed for the type of motility.^26^

### 2.9 Sensory Evaluation

Sensory evaluation of air-fried chicken breast chunks was performed by a trained panel of 10 panelists using a 9-point hedonic scale (1-point as extremely poor and 9-points as excellent) at a local meat processing plant located in Lahore, Pakistan. A sample assessment was done for various organoleptic characteristics such as color, flavor, taste, chewiness, juiciness, and overall acceptability. Samples were air-fried before 10 min of sensory evaluation and warmly served. Panelists were supplied with water for rinsing the oral cavity between the samples.^27^

### 2.10 Statistical Analysis

The acquired data were expressed as the mean values of three replicates, and standard deviations were analyzed statistically by estimating variance by applying SPSS version 25.0 (IBM, New York, NY, USA). One-way ANOVA and LSD’s *post-hoc* analysis were used for multiple comparisons. For all tests, *p*-values of *P* < 0.05 were considered statistically significant.

## 3 Results and Discussions

### 3.1 Physiochemical Analysis

The results of moisture content, protein content, lipid content, and pH of thawed samples and control samples are shown in (Table 3). Significant variations (*p* < 0.05) were observed in the moisture content of samples thawed using distinct techniques. Results showed that moisture content was highest in R_0_ (75.43%) and lowest in R_1_ (72.20%). The control sample depicted a higher percentage of moisture compared to other samples, followed by R_5_, which was subjected to oven thawing (74.07%). Moisture loss has been linked to the time required for thawing. The present findings are in line with those of Li et al.^15^ and Xia et al.,^16^ who reported an amplified moisture loss in chicken meat processing. The fat content of thawed samples showed non-significant (*P* > 0.05) variation, whereas protein content varied significantly as a result of thawing, which was in accordance with the study of Leygonie et al. (2012).^7^ In comparison to control (22.06%), the highest protein was retained by R_1_, which was 23.10%. The pH of thawed chicken fillets varied significantly (*p* < 0.05) from 5.94 to 6.08; a peak value (6.08) was detected in R_o_ because there was no storage of fresh meat samples. On the other hand, the lowest pH (5.94) was portrayed by R_4_, associated with enzymatic activity and microbial activity that promoted proteolytic reactions that consequently increased the release of the H+ ions and ultimately lowered the pH of thawed chicken samples as compared to the control group (R_0_). The present findings are in close relation with those of Duygu and Ümit,^28^ who studied the impact of different thawing methods (conventional, ohmic heating, and refrigeration) on meat and reported that pH varied in a range of 5.47–5.80 for a storage period of 6 months.

**Table 3:** Effect of Thawing Treatments on Physiochemical Parameters of Chicken Meat Fillets^a^

	variables			
treatments	moisture (%)	crude fat (%)	crude protein (%)	pH
R_0_	75.43^A^ ± 0.11	1.04^C^ ± 0.05	22.06^E^ ± 0.05	6.08^A^ ± 0.05
R_1_	72.20^D^ ± 0.10	1.08^A^ ± 0.02	23.10^A^ ± 0.04	5.98^CD^ ± 0.04
R_2_	73.83^C^ ± 0.05	1.07^B^ ± 0.02	22.40^C^ ± 0.04	6.01^B^ ± 0.03
R_3_	72.40^D^ ± 0.01	1.08^A^ ± 0.05	22.80^B^ ± 0.03	5.99^C^ ± 0.04
R_4_	72.33^D^ ± 0.04	1.08^A^ ± 0.01	22.77^B^ ± 0.05	5.94^E^ ± 0.04
R_5_	74.07^B^ ± 0.04	1.03^C^ ± 0.05	22.20^D^ ± 0.03	5.97^D^ ± 0.03

a Different superscripts in a row indicate a significant difference between the means (*p* < 0.05).

### 3.2 Protein Solubility

The protein solubility of thawed chicken meat fillets is illustrated in (Table 4). It is evident from the results shown in (Table 4) that protein solubility varied significantly (*P* < 0.05) from 298.27 to 238.63 mg/g for the different thawing treatments. The maximum solubility (298.28 mg/g) was observed in R_0_, while the minimum solubility (238.63 mg/g) was observed in R_1_. It can be concluded from the results that protein solubility decreased in all treatments of thawed samples in contrast to the control. Protein solubility is a significant indicator in determining the quality of chicken, and a decreased solubility is marked due to the oxidative deterioration of proteins.^9^ An increase in surface hydrophobicity results in lower protein solubility, which has been related to an increase in exudate during thawing.^29^ Our findings are in accordance with those of Zhang et al.^10^ who reported a similar effect on meat composition due to thawing.

**Table 4:** Effect of Thawing Treatments on Protein Solubility and Shear Force of Chicken Meat Fillets^a^

	variables	
treatments	protein solubility (mg/g)	shear force (N)
R_0_	298.26^A^ ± 0.02	7.95^F^ ± 0.05
R_1_	238.63^F^ ± 0.02	9.54^A^ ± 0.01
R_2_	247.63^E^ ± 0.03	9.28^B^ ± 0.02
R_3_	266.53^C^ ± 0.03	8.46^D^ ± 0.05
R_4_	262.77^D^ ± 0.04	9.04^C^ ± 0.01
R_5_	288.97^B^ ± 0.05	8.24^E^ ± 0.02

a Different superscripts in a row indicate significant difference between the means (*p* < 0.05).

### 3.3 Shear Force

Shear force is closely related to the tenderness and sensory attributes of the chicken. So, from a sensory perspective, it is regarded as one of the most important factors in assessing meat quality and storage stability. A significant (*P* < 0.05) variation was observed in shear force because of the thawing treatments, ranging from 7.95 to 9.54 N, as shown in (Table 4). The greatest shear force value was (9.54 N) presented by R_1,_ whereas R0 depicted the lowest value (7.95 N). The mean differences for R_0_, R_1,_ and R_2_ indicated significant (*P* < 0.05) differences. Compared with R_0_ shear force increased by 20.10, 16.90, 6.70, 13.88, and 6.30% for R_1_, R_2_, R_3_, R_4_, and R_5_ respectively. Similarly, Leygonie et al.^30^ reported an increase in the shear force of frozen ostrich samples after thawing, whereas Zhuang and Savage,^34^ reported a higher shear force in samples that were cooked directly from the frozen state as compared to thawed samples.

### 3.4 Cooking Loss and Thawing Loss

A significant variation was observed in the thawing loss of chicken meat fillets for each thawed treatment compared to control samples (R_0_), which is elucidated in (Table 5). The highest thawing loss (4.67%) was observed in R_1_, probably attributed to its extended thawing followed by microbial activity to disrupt protein structures, thus leading to enhanced moisture loss. On the other hand, the lowest thawing loss (2.10%) was noticed in R_5,_ possibly associated with decreased thawing span, enzymatic reactions, and microbial activity. It was evident that the oven-thawed samples (R_5_) were more identical to the fresh chicken fillets (R_0_) in terms of texture and color.

**Table 5:** Effect of Thawing Treatments on Cooking Loss and Thawing Loss of Chicken Meat Fillets^a^

	variables (%)	
treatments	cooking loss	thawing loss
R_0_	13.45^F^ ± 0.01	
R_1_	13.56^E^ ± 0.02	4.67^A^ ± 0.05
R_2_	14.22^B^ ± 0.01	2.67^D^ ± 0.05
R_3_	14.12^D^ ± 0.01	4.00^B^ ± 0.02
R_4_	14.27^A^ ± 0.02	4.20^C^ ± 0.02
R_5_	14.16^C^ ± 0.02	2.10^E^ ± 0.02

a Different superscripts in a row indicate a significant difference between the means (*p* < 0.05).

Furthermore, freezing and thawing alter the mineral and protein content of muscles, due to which thawing loss varies for every thawing technique. The present findings agree with Leygonie et al.,^30^ who reported an increased drip loss in frozen-thawed ostrich samples. Differences in thawing loss for various techniques are interlinked with modifications in the muscle and moisture content of meat.

The cooking loss of chicken fillets was not significantly (*P* > 0.05) affected by the duration and temperature of various thawing techniques, as depicted in Table 5. The cooking loss varied from 13.45 to 14.27% for the various thawing treatments. The highest value (14.27%) was observed in R_4_, whereas the lowest value (13.45%) was in R_0_ (control group). Similarly, Vieira et al.,^31^ reported a non-significant impact of various thawing techniques on the cooking loss of meat fillets due to the chemically bound water that is removed because of heat treatments.

### 3.5 Lipid and Protein Oxidation

Lipid oxidation is closely related to the quality of chicken meat as well as to the economic sustainability of the meat industry. So, from the point of view of quantity, it is one of the most critical factors used to calculate the exact quality and storage stability of chicken meat or its products. The data regarding the mean values of lipid oxidation in chicken breast fillets are explicated in (Table 6). It is evident from the results presented in (Table 6) that the MDA values of thawed chicken breast fillets varied in a significant manner (*p* < 0.05) from 0.74 mg MDA/Kg to 1.15 mg MDA/Kg for the different thawing treatments. The highest value (1.15 mg MDA/Kg) was recorded for R_2_, whereas the lowest value (0.74 mg MDA/Kg) for R_0_ (control group). The R_1_ sample showed a lipid oxidation content comparatively higher than the control sample (0.94 mg MDA/Kg). Samples R_3_ (0.79 mg MDA/Kg) and R_5_ (0.78 mg MDA/Kg) showed similar results for lipid oxidation with non-significant variations.

**Table 6:** Effect of Thawing Treatments on Lipid Oxidation and Protein Oxidation of Chicken Meat Fillets^a^

	variables (%)	
treatments	lipid oxidation TBARS (mg MDA/Kg)	protein oxidation (carbonyl content nM/mg protein)
R_0_	0.74^E^ ± 0.02	0.99^E^ ± 0.01
R_1_	0.94^B^ ± 0.01	1.08^C^ ± 0.01
R_2_	1.15^A^ ± 0.02	1.21^A^ ± 0.01
R_3_	0.79^D^ ± 0.02	1.13^B^ ± 0.04
R_4_	0.90^C^ ± 0.02	1.12^BD^ ± 0.02
R_5_	0.78^D^ ± 0.03	1.02^DE^ ± 0.04

a Different superscripts in a row indicate a significant difference between the means (*p* < 0.05).

The carbonyl content, an indicator of protein oxidation in thawed chicken breast fillets, is explicated in Table 6. The results elucidated in (Table 6) showed that carbonyl content varied significantly (*P* < 0.05) from 0.99 nM/mg protein to 1.21 nM/mg protein for the various thawing treatments. R_2_ presented a maximum protein oxidation (1.21 nM/mg protein), whereas a minimum oxidation (0.99 nM/mg protein) was recorded for the control group (R_0_). The study of Xiong^32^ also reflected a low carbonyl content in the control group and a high carbonyl content for water immersion thawing. Furthermore, protein and lipid oxidation usually disrupt the organizational structure in the muscles and reduce the water-holding capacity and gelling properties, as reported by Xiong^32^ and Rowe et al.^33^

### 3.6 Microbial Evaluation

The TPC of thawed chicken breast fillets is elucidated in (Table 7). TPC varied significantly (*P* < 0.05) from 11.00 × 10^3^ to 29.00 × 10^3^ CFU/g for the different thawing treatments. The peak value (27.00 × 10^3^ CFU/g) was recorded for R_1_, while R_0_ presented the lowest TPC (11.00 × 10^3^ CFU/g). Schlisselberg et al.^35^ reported a similar trend for TPC in several thawing techniques. It must be remembered that the total viable count is closely related to the time and temperature at which various samples are thawed. *Coliforms* and *L. monocytogenes* should be negative in all meat or other edible products as per FDA circular guidelines,^36^ and in the present study, both pathogens were absent for all thawing treatments, as portrayed in (Table 7).

**Table 7:** Effect of Thawing Treatments on Microbiological Evaluation of Chicken Meat Fillets^a^

	variables (%)		
treatments	TPC (103CFU/g)	*coliform* count	listeria count
R_0_	11.00^E^ ± 0.03	ND	ND
R_1_	27.00^B^ ± 0.04	ND	ND
R_2_	29.00^A^ ± 0.02	ND	ND
R_3_	22.33^C^ ± 0.03	ND	ND
R_4_	18.33^D^ ± 0.02	ND	ND
R_5_	14.33^D^ ± 0.04	ND	ND

a Different superscripts in a row indicate a significant difference between the means (*p* < 0.05). ND = not detected.

### 3.7 Sensory Evaluation

Organoleptic characteristics are considered prominent factors in determining meat quality. The effect of various thawing treatments on sensory attributes of chicken meat fillets was assessed and compared to R_0_ (control group) to suggest the thawing treatment with the minimum sensory losses. The statistical findings showed that all sensory parameters (color, flavor, chewiness, taste, and overall acceptability) varied non-significantly (*P* > 0.05) except juiciness because of thawing loss.

**Figure 1:**
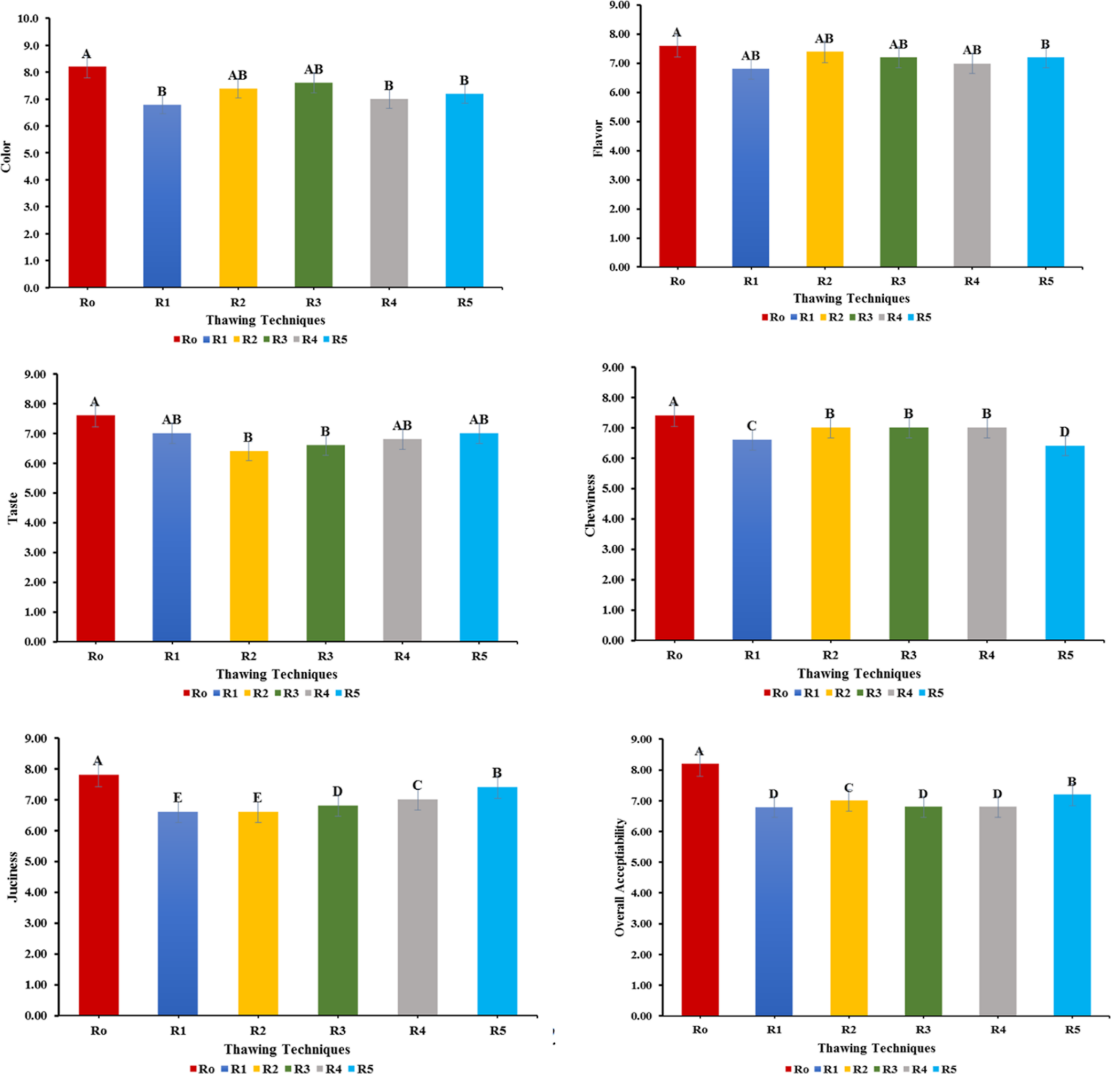
Effect of different thawing treatments on the organoleptic characteristics of chicken meat fillet. Columns labeled with different letters are significantly different, *p* < 0.05 (*n* = 3).

The control sample (R_0_) scored highest (8.2) for overall acceptability; however, R_5_ (oven thawing) scored highest (7.20) in all thawed samples, followed by R_2_ with 7.0 scores. The fresh meat fillets (R_0_) received the highest scores (7.80) by the panel for juiciness. On the other hand, among thawed samples, oven-thawed samples showed higher juiciness (7.40), followed by R_4_, R_3_, and R_2_ with sensory scores of 7.0, 6.80, and 6.60, respectively (Figure 1).

## 4 Conclusions

The effects of various thawing treatments were monitored on the quality, microbiota, and organoleptic properties of chicken meat fillets. The results of the present study showed that quality indicators were negatively affected by the freezing and subsequent thawing of the chicken meat fillets. Lipid and crude protein contents increased because of the higher thawing loss. Shear force, thawing loss, and protein and lipid oxidation increased due to diminished protein structure, which is incapable of holding water, whereas pH and protein solubility decreased. However, the combined thawing and oven thawing showed better results. The TPC was increased due to increased time for microbial proliferation at higher temperatures. Cooking loss was negligible as it reduced the chemically bound water instead of the physically bound water. The sensory properties of the chicken meat were negatively affected compared to the control samples. This may probably be attributed to a reduced moisture content; however, R_3_ and R_5_ were closer to R_0_. Hence, R_3_ and R_5_ are suggested as the best thawing treatments in all respects; however, oven thawing (R_5_) cannot be adopted for commercial applications. Further research is required to optimize the oven thawing treatment for the chicken meat industry.
